# Dominant RP in the Middle While Recessive in Both the N- and C-Terminals Due to *RP1* Truncations: Confirmation, Refinement, and Questions

**DOI:** 10.3389/fcell.2021.634478

**Published:** 2021-02-19

**Authors:** Junwen Wang, Xueshan Xiao, Shiqiang Li, Panfeng Wang, Wenmin Sun, Qingjiong Zhang

**Affiliations:** State Key Laboratory of Ophthalmology, Zhongshan Ophthalmic Center, Sun Yat-sen University, Guangzhou, China

**Keywords:** *RP1*, retinitis pigmentosa, exome sequencing, variants, inheritance pattern

## Abstract

*RP1* truncation variants, including frameshift, nonsense, and splicing, are a common cause of retinitis pigmentosa (RP). *RP1* is a unique gene where truncations cause either autosomal dominant RP (adRP) or autosomal recessive RP (arRP) depending on the location of the variants. This study aims to clarify the boundaries between adRP and arRP caused by *RP1* truncation variants based on a systemic analysis of 165 *RP1* variants from our in-house exome-sequencing data of 7,092 individuals as well as a thorough review of 185 *RP1* variants from published literature. In our cohort, potential pathogenic variants were detected in 16 families, including 11 new and five previously described families. Of the 16, seven families with adRP had heterozygous truncations in the middle portion, while nine families with either arRP (eight) or macular degeneration had biallelic variants in the N- and C-terminals, involving 10 known and seven novel variants. In the literature, 147 truncations in *RP1* were reported to be responsible for either arRP (85) or adRP (58) or both (four). An overall evaluation of *RP1* causative variants suggested three separate regions, i.e., the N-terminal from c.1 (p.1) to c.1837 (p.613), the middle portion from c.1981 (p.661) to c.2749 (p.917), and the C-terminal from c.2816 (p.939) to c.6471 (p.2157), where truncations in the middle portion were associated with adRP, while those in the N- and C-terminals were responsible for arRP. Heterozygous truncations alone in the N- and C- terminals were unlikely pathogenic. However, conflict reports with reverse situation were present for 13 variants, suggesting a complicated pathogenicity awaiting to be further elucidated. In addition, pathogenicity for homozygous truncations around c.5797 and thereafter might also need to be further clarified, so as for missense variants and for truncations located in the two gaps. Our data not only confirmed and refined the boundaries between dominant and recessive *RP1* truncations but also revealed unsolved questions valuable for further investigation. These findings remind us that great care is needed in interpreting the results of *RP1* variants in clinical gene testing as well as similar features may also be present in some other genes.

## Introduction

*RP1* (OMIM 603937), mapped to chromosome 8q11.2–12.1, is an axonemal microtubule-associated gene with four exons, where the protein-coding region is in the last three (Blanton et al., 1991; Pierce et al., 1999; Méndez-Vidal et al., 2014). It encodes a 2,156-amino acid protein that is specifically expressed in photoreceptor connecting cilia (Liu et al., 2002, 2003), participating in protein transport between the inner and outer segments of the photoreceptors via two domains: the doublecortin (DCX) domain and the bifocal (BIF) domain (Pierce et al., 1999; Liu et al., 2002; Siemiatkowska et al., 2012; Méndez-Vidal et al., 2014).

Variants in *RP1* were initially identified to be responsible for autosomal dominant retinitis pigmentosa (adRP) and later for autosomal recessive retinitis pigmentosa (arRP) (OMIM 180100) (Pierce et al., 1999; Sullivan et al., 1999; Khaliq et al., 2005). *RP1* is listed as the top seventh of the most frequently implicated genes in inherited retinal disease based on a large cohort (Pontikos et al., 2020). To date, a large number of potential pathogenic variants in *RP1* have been reported, demonstrating a unique correlation between mutation location and pattern of inheritance. Usually, heterozygous truncation variants in the middle portion contributed to adRP, while biallelic truncation variants in the N- and C- terminals were associated with arRP. The boundaries between dominant and recessive variants have been suggested in previous studies but conflict reports were also present (Chen et al., 2010; Al-Rashed et al., 2012; Siemiatkowska et al., 2012; Eisenberger et al., 2013; El Shamieh et al., 2015; Kabir et al., 2016; Nanda et al., 2019; Riera et al., 2020). It is expected to confirm and refine the boundaries as well as the genotype–phenotype correlation of *RP1* variants based on a large dataset, especially at the era of widespread application of clinical genetic testing.

In this study, *RP1* variants were selected and analyzed based on our in-house exome-sequencing data from 7,092 individuals with different forms of eye conditions. *RP1* variants in published literature were systematically reviewed. These data further confirmed and refined the boundaries of *RP1* truncation variants, in which adRP associated with heterozygous variants in the middle portion, while arRP associated with biallelic variants in the N- and C- terminals. Besides, conflict reports in a reverse situation may call attention and be studied further. In clinical gene testing, pathogenicity of individual truncation variants in *RP1* might be complicated and should be explained with great care, especially for novel variants as well as those variants with conflict consequence.

## Materials and Methods

### Subjects

Individuals with various forms of eye conditions were collected by our team based on our ongoing program on genetic study of inherited eye diseases. Prior to their participation, written informed consent adhering to the tenets of the Declaration of Helsinki was obtained from participants or their guardians. Clinical data and peripheral venous blood samples were collected from these individuals and their available family members. Genomic DNA was extracted from the leukocytes of peripheral blood based on procedures as described in our previously study (Wang et al., 2010). This study was approved by the institutional review board of the Zhongshan Ophthalmic Center.

### *RP1* Variant Identification From Our In-House Data

Exome sequencing was performed on genomic DNA samples from the 7,092 individuals, including whole-exome sequencing (WES) on 5,307 and targeting exome sequencing (TES) on 1,785. The procedures used to perform WES and TES were described in detail in our previous studies (Li et al., 2015; Wang et al., 2019).

*RP1* variants were collected from exome-sequencing data of 7,092 individuals with various forms of eye conditions, including 1,019 with RP, 1,217 with glaucoma, 1,299 with high myopia, 492 normal controls, and 3,065 with other eye conditions. *RP1* variants were initially filtered following the procedures described in our previous studies (Jiang et al., 2014; Li et al., 2015). The candidate variants in *RP1* were then annotated as the following steps: (1) the allelic frequency of each variant was annotated according to the gnomAD database^1^; (2) missense variants were predicted using five *in silico* online tools, including REVEL^2^, CADD^3^, SIFT^4^, PolyPhen2^5^, and PROVEAN^6^; (3) the splicing effect of variants in the intronic region as well as synonymous variants were predicted using the Berkeley Drosophila Genome Project (BDGP^7^); (4) genotype–phenotype correlation was used to identify potential pathogenic variants. Sanger sequencing was used to validate potential pathogenic variants and segregation analysis in available family members was carried out to further validate the pathogenicity. The primers used herein were designed using the Primer3 online tool^8^.

### Literature Review of *RP1* Variants

The “RP1” was used as the keyword to search PubMed^9^, Google Scholar^10^, and The Human Gene Mutation Database (HGMD^11^) on September 1, 2020. The *RP1* variants were collected from these resources and annotated as noted in the above section. The phenotypes associated with these *RP1* variants are summarized.

### Statistical Analysis

All statistical analyses were performed by using SPSS software version 25.0 (Armonk, NY: IBM Corp). The difference of proportion of macular abnormalities between the two groups of patients with adRP and arRP was analyzed using the Chi-square test. The difference of the frequency of truncation variants between in-house exome sequencing data and gnomAD database was tested via the Chi-square test. A Mann–Whitney *U*-test was used to determine the phenotypic differences between the two groups of patients because the data were not distributed normally. A *P*-value of less than 0.05 was considered statistically significant.

## Results

### *RP1* Variants Detected in Our In-House Data

Totally, 165 variants were detected based on our exome sequencing data, including 143 missense variants, 20 truncation variants (10 nonsense, nine frameshift, and one splicing change variants), and two inframe variants. Potential pathogenic variants were detected in 16 families, including 11 new families and five previously described families (Xu et al., 2014; Wang et al., 2019). Of the 16 families, seven families with adRP had heterozygous truncation variants in the middle portion, while eight families with arRP and one family with macular degeneration (MD) had biallelic variants in the N- and C-terminals, involving 17 variants (Table 1 and Supplementary Table S1). Of the 17 variants, seven were novel, i.e., c.256C > A (p.Pro86Thr), c.1987A > T (p.Lys663^∗^), c.2062G > T (p.Gly688^∗^), c.2399_2400del (p.Lys800Serfs^∗^6), c.2700dup (p.Pro901Thrfs^∗^2), c.5017del (p.Tyr1673Metfs^∗^37), and c.6341_6343del (p.Ser2114del). These variants were confirmed by Sanger sequencing and co-segregated with the disease in families with available family members (Supplementary Figure S1). Biallelic missense variants were detected in five probands, in which one was with adRP, while the other four were with other conditions (Supplementary Table S2).

**TABLE 1:** Potential pathogenic variants associated with retinitis pigmentosa (RP) in our cohort.

Position	Exon	Nucleotide change	Effect	Status	Family number	Family ID	Allele in	HGMD	References
		(NM_006269.1)			New + Reported^#^	(Reported^#^)	gnomAD		
**1. Single heterozygous variants**									
5,5538,429	4	c.1987A > T	p.Lys663*	Het	1 + 0	20,455	/	/	Novel
5,5538,471	4	c.2029C > T	p.Arg677*	Het	2 + 0	7,948, 18,926	/	DM	Guillonneau et al., 1999; Pierce et al., 1999; Martin-Merida et al., 2018
5,5538,504	4	c.2062G > T	p.Gly688*	Het	1 + 0	9,053	/	/	Novel
5,5538,558	4	c.2117del	p.Gly706Valfs*7	Het	0 + 1	(4,293)	/	DM	Xu et al., 2014
5,5538,841	4	c.2399_2400del	p.Lys800Serfs*6	Het	1 + 0	12,426	/	/	Novel
5,5539,142	4	c.2700dup	p.Pro901Thrfs*2	Het	1 + 0	18,611	/	/	Novel
**2. Biallelic variants**									
5,5533,782	2	c.256C > A	p.Pro86Thr	ComHet	1 + 0	12,349	22/282,760	/	Novel
5,5533,779	2	c.257dup	p.Arg87Serfs*48	ComHet	0 + 1	(14,948)	DM	/	Huang et al., 2015; Wang et al., 2019
5,5533,952	2	c.426dup	p.Ala143Serfs*86	ComHet	0 + 1	(6,170)	/	DM	Xu et al., 2014
5,5534,133	2	c.607G > C	p.Gly203Arg	ComHet	0 + 1	(6,170)	/	DM	Xu et al., 2014
5,5538,558	4	c.2116G > C	p.Gly706Arg	ComHet	0 + 1	(13,159)	89/282,438	DM	Wang et al., 2019
5,5540,748	4	c.4306del	p.Ser1436Profs*16	ComHet	0 + 1	(13,159)	/	DM	Wang et al., 2019
5,5541,132	4	c.4690del	p.Val1564*	Hom	3 + 0	6,609, 21,210, 21,311	/	DM	Wang et al., 2019
5,5541,246	4	c.4804C > T	p.Gln1602*	ComHet	0 + 1	(14,948)	3/251,080	DM	Avila-Fernandez et al., 2012; Ezquerra-Inchausti et al., 2018; Wang et al., 2019
5,5541,459	4	c.5017del	p.Tyr1673Metfs*37	Hom	1 + 0	8,089	/	/	Novel
5,5542,239	4	c.5797C > T	p.Arg1933*	Hom	0 + 1	(13,685)	49/281,934	DM?	Yeung et al., 2001; Fujinami et al., 2016; Li et al., 2018; Wang et al., 2019
5,5542,783	4	c.6341_6343del	p.Ser2114del	ComHet	1 + 0	12,349	10/280,914	/	Novel

*^#^Families that have been reported by us previously. The genome build for these chromosomal positions was UCSC GRCh37/hg19. RP, retinitis pigmentosa; Het, heterozygous; Hom, homozygous; ComHet, compound heterozygous; DM, disease-causing mutations.*

In addition, 12 single heterozygous truncation variants were identified in 44 individuals from our cohort. Of the 12 variants, 11 located at the N- and C- terminals were identified in 43 unrelated individuals and were considered non-pathogenic. Of the 43 individuals, seven were affected with RP and the remaining 36 with various conditions other than RP (Supplementary Table S3). Five different truncations involved in the seven patients with RP were unlikely pathogenic based on the following evidence: (1) the c.257dup (p.Arg87Serfs^∗^48) was detected in three patients with RP and four individuals with unrelated conditions; (2) the c.1826C > G (p.Ser609^∗^) was detected in one patient with RP who had biallelic pathogenic variants in *EYS*; (3) the c.4690del (p.Val1564^∗^) was detected in a patient with X-linked RP and a patient with other condition; (4) the c.5017del (p.Tyr1673Metfs^∗^37) was detected in one patient with isolated RP and four patients with other conditions; and (5) the c.5797C > T (p.Arg1933^∗^) was detected in one patient with RP and 19 unrelated individuals with other conditions. These heterozygous variants were not enriched in patients with RP, and their frequency in our cohort is comparable with that in the East Asian population in gnomAD database (*P* = 0.94), suggesting that these 11 variants were unlikely pathogenic for retinal degeneration in heterozygous status. Apart from the 11, the remaining heterozygous c.2391_2392del (p.Asp799^∗^) variant was located in the middle portion of *RP1* and was detected in a college student with late-onset high myopia without any sign of RP. The p.Asp799^∗^ is a known variant associated with cone-rod dystrophy (CRD) in homozygous status in a previous study (El Shamieh et al., 2015). Besides, none of the heterozygous missense variants predicted to be damaging were associated with adRP in our cohort.

### *RP1* Variants Reported in the Literature

A total of 185 *RP1* variants have been reported in the literature, including 147 truncation variants (51 nonsense, 95 frameshift, and one splicing) (Supplementary Table S4) and 38 missense variants (Supplementary Table S5). Of the 147 truncation variants, 85 were reported to cause recessive diseases, 58 were reported to lead to dominant diseases, and four were identified in patients with both dominant and recessive diseases. Of the 38 missense variants, 22 were reported to be recessive, 15 were reported to be dominant, and one was reported to be both dominant and recessive (Supplementary Table S5). The diseases associated with the 185 variants of *RP1* included RP, CRD, Leber congenital amaurosis (LCA), MD, and unclassified inherited retinal dystrophy (IRD) (Supplementary Tables S4, S5).

### The Location of *RP1* Truncation Variants

**FIGURE 1:**
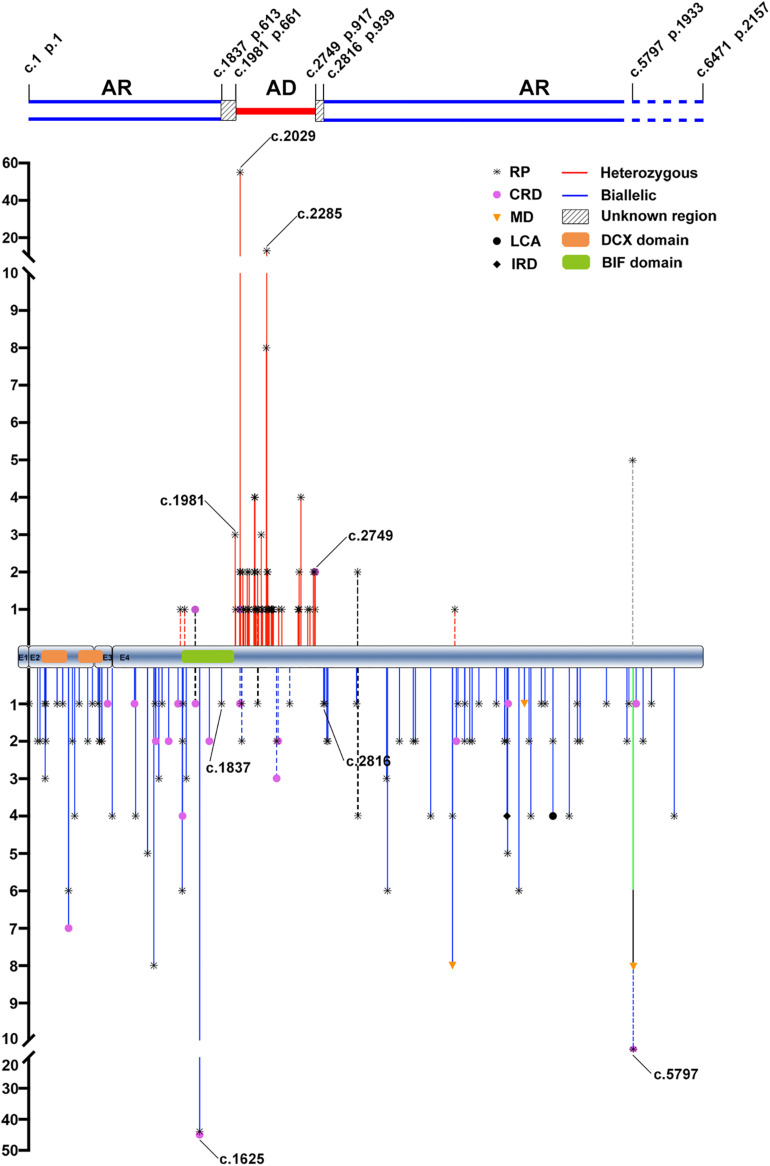
The distribution and frequency of the potential pathogenic truncation variants in *RP1* identified in the present and previous studies. AR, autosomal recessive; AD, autosomal dominant; RP, retinitis pigmentosa; CRD, cone-rod dystrophy; MD, macular degeneration; LCA, Leber congenital amaurosis; IRD, inherited retinal disease. The positions and allele counts of the heterozygous *RP1* variants are displayed above, while those of the biallelic *RP1* variants are displayed below. The two blue lines above represent the autosomal recessive retinitis pigmentosa (arRP) region, the single red line represents the autosomal dominant retinitis pigmentosa (adRP) region, and the diagonal line between them represents the unknown regions. The two blue dashed lines are used to indicate the pathogenicity of homozygous truncations around the c.5797, and thereafter, need to be further clarified. DCX domain: c.106–354 (p.36–188) and c.460-699 (p.154–233). BIF domain: c.1456–1959 (p.486–653).

In our cohort, the six *RP1* heterozygous truncation variants associated with adRP in seven families were located in the region from c.1987 (p.663) to c.2700 (p.901), which was immediately downstream of the BIF domain (Table 1 and Figure 1). For the nine families with biallelic variants, five had homozygous truncation variants located in the C-terminal region, including c.4690del (p.Val1564^∗^) in three families, c.5017del (p.Tyr1673Metfs^∗^37) in one, and c.5797C > T (p.Arg1933^∗^) in one; one had compound heterozygous truncations, c.257dup (p.Arg87Serfs^∗^48) and c.4804C > T (p.Gln1602^∗^); two had compound heterozygous variants, one truncation and one missense; the remaining one had compound heterozygous variants, one inframe and one missense (Table 1 and Supplementary Table S1). In addition, 11 likely benign single heterozygous truncation variants were located at N- and C- terminals (Supplementary Table S3).

From the literature, 58 heterozygous truncation variants of *RP1* were reported to be associated with dominant diseases. Of the 58, 55 from 152 families were located in a region from c.1981 (p.661) to c.2749 (p.917) (Figure 1). Of the 85 biallelic variants from the literature, 79 from 117 families were located in the N- and C-terminal regions, namely, c.1 (p.1)–c.1837 (p.613) and c.2816 (p.939)–c.6471 (p.2157) (Figure 1). Combining our in-house data and the data from the literature, three regions in *RP1* were suggested, N-terminal from c.1 (p.1) to c.1837 (p.613), middle portion from c.1981 (p.661) to c.2749 (p.917), and C-terminal from c.2816 (p.939) to c. 6471 (p.2157) (Figure 1). A common feature could be identified as follows: truncations in the middle portion are associated with adRP, while those in the N- and C- terminals are responsible for arRP, which was supported by most truncation variants (91.9%, 147/160).

However, conflict results were reported for at least 13 truncation variants in *RP1* (Jacobson et al., 2000; Payne et al., 2000; Baum et al., 2001; Xiaoli et al., 2002; Kawamura et al., 2004; Eisenberger et al., 2013; Sullivan et al., 2013; El Shamieh et al., 2015; Yoon et al., 2015; Carrigan et al., 2016; Ellingford et al., 2016; Huang et al., 2017; Van Cauwenbergh et al., 2017; Li et al., 2018; Martin-Merida et al., 2019; Nikopoulos et al., 2019; Verbakel et al., 2019; Huckfeldt et al., 2020; Supplementary Table S6). Of the 13 variants, seven located in the middle portion were reported to be responsible for arRP rather than adRP, while six located in the N- and C-terminals caused adRP rather than arRP. Surprisingly, four of the 13 were involved in both adRP and arRP (Supplementary Table S6). In addition, the c.2391_2392del (p.Asp799^∗^) located in the middle portion was reported to cause arCRD in homozygous status, which was supported by our study where a heterozygous carrier did not have any sign of RP. These raise questions on how to explain the common feature vs. the rare conflict results.

### The Missense Variants in *RP1*

For the 38 missense variants from the literature, 22 variants involved in recessive retinal degeneration, and all of them were located at the N- and C-terminals (Supplementary Figure S2). The c.606C > A (p.Asp202Glu) variant was the most common and was detected in 17 families in homozygous status, including 13 families with arMD (Huckfeldt et al., 2020; Riera et al., 2020), three families with arRP (Aldahmesh et al., 2009; Huckfeldt et al., 2020), and one family with arCRD (Huckfeldt et al., 2020). This variant was absent from the gnomAD database. In contrast, 15 missense variants were reported to cause adRP and distributed scattered across the whole coding region of *RP1*, but segregation information was not described for 13 of the 15 variants. The remaining one, c.1118C > T (p.Thr373Ile), with a frequency of 3434/282692 in gnomAD database, was reported in patients with either dominant or recessive retinal degeneration, which is apparently a non-pathogenic variant. No heterozygous missense variant predicted to be damaging was identified to be responsible for adRP in our cohort.

### Genotype–Phenotype Correlation of *RP1* Variants in our In-House Data and the Literature

**FIGURE 2:**
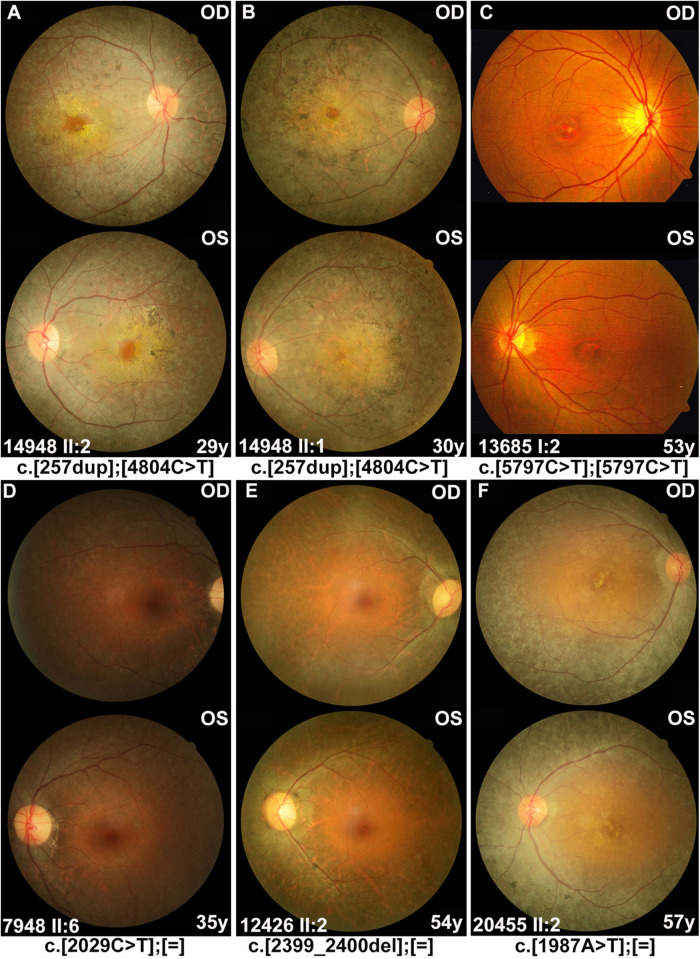
Fundus photographs of the affected individuals with *RP1* variants. **(A,B)** Severe phenotypes were shown in patients with biallelic *RP1* variants, including waxy pale optic disc, attenuated vessels, and periphery degeneration with bone spicule pigmentation and involving macular region. **(C)** A 53 year-old patient diagnosed with macular degeneration rather than RP, who carried homozygous variant c.5797C > T (p.Arg1933*) in our cohort. **(D–F)** Patients with heterozygous *RP1* variants were characterized by peripheral pigment disorders and scattered distribution of bone spicule pigmentation, both of which were often absent in the macular region.

**FIGURE 3:**
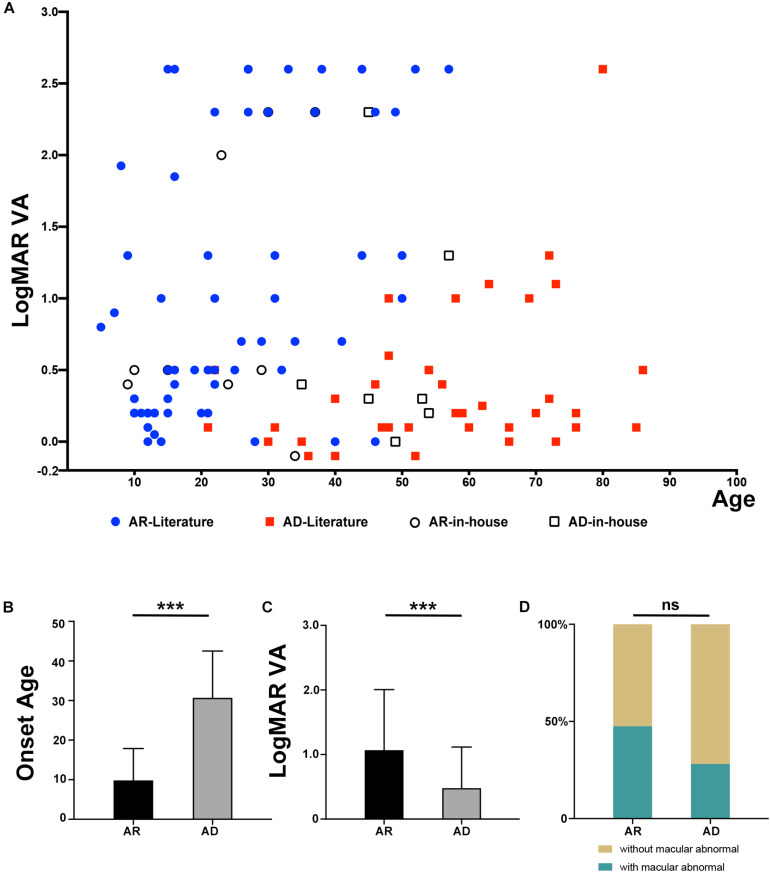
Comparison of phenotypes in patients with an inheritance pattern for arRP and adRP. **(A)** Scatter plots of the LogMAR visual acuity of patients with AR and AD inheritance from our in-house data and the previously published literature. **(B)** Comparison of the onset ages of patients with AR and AD inheritance from our in-house data and the previously published literature. ***P = 2.76*10^–11^. **(C)** Comparison of the LogMAR visual acuity of patients with AR and AD inheritance from our in-house data and the previously published literature. ***P = 1.75*10^–4^. **(D)** Comparison of the macular abnormalities, including macular degeneration or macular atrophy, of patients with AR and AD inheritance from our in-house data and the previously published literature. ns mean not significant (*P* = 0.061).

From our in-house data, a total of 21 individuals from 16 families were detected with potential pathogenic truncation variants in *RP1*. Clinical data were available in 17 of the 21 individuals. These individuals complained of a variety of initial clinical manifestation, including night blindness, decreased visual acuity, or narrowing of visual field. The age at onset of these individuals ranged from childhood to 52 years old. The age at examination ranged from 9 to 57 years with visual acuity varying from no light perception to 0.5 (Snellen equivalent). Sixteen of the 17 individuals with potential pathogenic variants showed typical RP fundus changes including waxy pale optic disc, attenuated vessels, and periphery degeneration with bone spicule pigmentation with or without obvious macula involvement (Figure 2). Electroretinogram recordings of four patients showed severely decreased to distinguished responses for both of the rods and cones at the ages of 10, 34, 37, and 53 years old, respectively. The remaining one of the 17, a 53 year-old patient with a homozygous c.5797C > T (p.Arg1933^∗^) had macular degeneration rather than RP (Figure 2C). Combined with our in-house data and the data obtained from the literature review (Figure 3), patients with arRP due to biallelic *RP1* variants had a significantly earlier age at onset (Figure 3B, *Z* = −6.66, *P* = 2.76^∗^10^–11^), worse visual acuity (Figure 3C, *Z* = −3.75, *P* = 1.75^∗^10^–4^), and seemingly more likely to have degeneration involving both of the macular and mid-peripheral retina (Figures 2, 3D, *P* = 0.061) (compared to adRP due to heterozygous *RP1* variants, in which mid-peripheral retina was mainly affected).

## Discussion

In this study, results from a systemic analysis of *RP1* variants from our in-house data as well as those from published literature demonstrate some common features of pathogenic variants in this gene, including: (1) about 80% of pathogenic variants are truncation variants; (2) truncation variants in the middle portion (c.1981 to c.2749) are associated with adRP, while those in the N-terminal (c.1 to c.1837) and C-terminal (c.2816 to c.6471) are responsible for arRP, supported by 91.9% of truncations; and (3) heterozygous truncation variants alone in the N- and C- terminals are unlikely pathogenic. Several questions remain to be clarified in future studies regarding the pathogenicity of the following *RP1* variants: (1) truncation variants located in the two gaps between the N-terminal and middle portion as well as between the middle portion and C-terminal; (2) homozygous truncation variants around the c.5797 and thereafter; (3) missense variants, especially those with adRP; (4) the mechanism for the common features of *RP1* truncation variants; and (5) the possible reasons for 8.1% of truncation variants with phenotypes contrary to the common feature. Awareness of these common features and unsolved questions is important in this era of widespread application of clinical gene testing.

The pathogenicity of truncation variants of *RP1* has been previously reported to be related to the location of the gene. Chen et al. (2010) first defined four classes of truncation variants in *RP1*: Class I, from p.1 to p.263, does not cause RP; Class II, from p.500 to p.1053, causes adRP; Class III, from p.263 to p.500 and from p.1053 to p.1751, causes arRP; Class IV, from p.1816 to p.2156, does not cause RP. However, variants in the non-pathogenic Class I and Class IV regions have subsequently been identified in patients with arRP (Avila-Fernandez et al., 2012; Eisenberger et al., 2013; Xu et al., 2014; Bravo-Gil et al., 2016; Kabir et al., 2016; Perez-Carro et al., 2016; Verbakel et al., 2019; Riera et al., 2020; Silva et al., 2020). In addition, some variants in the Class II region, associated with adRP, have been reported to cause arRP in homozygous or compound heterozygous status (Avila-Fernandez et al., 2012; Corton et al., 2013; Bravo-Gil et al., 2017; Ezquerra-Inchausti et al., 2018; Li et al., 2018; Martin-Merida et al., 2019; Silva et al., 2020). Therefore, several studies reported that the *RP1* variants at N-terminal and C-terminal regions were associated with arRP and those at the middle region resulted in adRP (Kabir et al., 2016; Nanda et al., 2019), and Nanda et al. (2019) suggested that the boundary of the region associated with adRP was located between p.677 and p.917. In our cohort, the c.257dup (p.Arg87Serfs^∗^48) variant, located in the Class I region, was identified in two siblings from one family with typical RP changes in trans with the c.4804C > T (p.Gln1602^∗^) (Figures 2A,B). Furthermore, the boundaries of these three regions were refined based on a systemic analysis of our in-house exome-sequencing data and the literature review, namely, the arRP region, from c.1 (p.1) to c.1837 (p.613) in the N-terminal and from c.2816 (p.939) to c.6471 (p.2157) in the C-terminal, and the adRP region, from c.1981 (p.661) to c.2749 (p.917) in the middle portion. The difference of the middle portion for adRP between this study and the previous studies is the extension of this region between p.677 and p.917 to p.661–p.917. The c.1981G > T (p.Glu661^∗^) variant is defined as the upstream boundary of the middle portion because it has been identified in three independent families with adRP and cosegregated with adRP in one family (Fernandez-San Jose et al., 2015; Martin-Merida et al., 2018; Riera et al., 2020). Although the boundaries among different regions have been well outlined based on our data and data from at least 291 families, there are still two gaps in between without enough information. As for the 13 truncation variants contrary to the common feature, some might be problematic while the others may represent a variable expression of phenotypes. For example, four truncation variants were initially reported to cause dominant retinal degeneration and then were reported to cause recessive diseases in trans with the other allele in subsequent studies. Clinically variable expression from hardly identifiable to typical phenotypes have been observed in RP patients from the same family with the same mutation in other gene like *RHO* (Luo et al., 2020), and such phenomenon could not be excluded for *RP1* variants. In addition, age-dependent expression of the diseases might also be considered. In these cases, wide-field examination of fundus such as scanning laser ophthalmoscope and electroretinogram may be of help in identifying mild or early signs of RP, especially in those carriers of individuals with variants associated with both dominant and recessive retinal degeneration. Co-segregation of those variants as well as well-defined phenotypes in family members may provide additional information in clarifying these conflict results. Moreover, a few variants with conflict result may represent a unique point-dependent rather than region-dependent pathogenicity of dominant or recessive nature. For example, the c.2391_2392del (p.Asp799^∗^), located in the middle portion and supposed to be causative for adRP, has been reported to cause arCRD with firm evidence in previous study (El Shamieh et al., 2015) and is identified in an adult without any sign of RP in heterozygous status in our cohort.

Besides, the c.5797C > T (p.Arg1933^∗^) variant located at the C-terminal has been reported to be non-pathogenic either in heterozygous or in homozygous status (Baum et al., 2001; Xiaoli et al., 2002; Nikopoulos et al., 2019). However, it can cause recessive diseases in trans with another truncation variant of *RP1* located upstream (Li et al., 2018; Nikopoulos et al., 2019; Verbakel et al., 2019). It has been suggested that the effect of the c.5797C > T (p.Arg1933^∗^) variant might be between monogenic and complex diseases (Baum et al., 2001; Xiaoli et al., 2002; Nikopoulos et al., 2019). In our cohort, this variant in homozygosis was identified in a 53 year-old singleton case with macular degeneration (Figure 2C). No pathogenic variants in other genes were detected in this patient based on the whole exome sequencing. Unfortunately, further clinical examination of the patients is unavailable except for fundus photographs. This raises questions on whether the c.5797C > T variant as well as other truncations downstream are pathogenic or not in homozygous status.

So far, it is unclear for the molecular mechanism about dominant in the middle while recessive in the N- and C- terminals for *RP1* truncation variants. Mutations in several other genes such as *GUCY2D* (Sharon et al., 2018), *RHO* (Luo et al., 2020), *CRX* (Yi et al., 2019), etc., are also associated with both dominant and recessive retinal degeneration, but the situation is a little different for them. For truncations in *CRX* and *RHO*, loss-of-function mutations are responsible for autosomal recessive diseases while dominant-negative mutations lead to autosomal dominant diseases (Yi et al., 2019; Luo et al., 2020). As for *GUCY2D*, most variants are associated with autosomal recessive LCA but the heterozygous substitution of the arginine at p.838 could cause autosomal dominant CRD (Sharon et al., 2018). The arginine at p.838 is the most sensitive position of GUCY2D protein. The mutants at arginine 838 shift the Ca^2+^-sensitivity in the guanylate cyclase-activating proteins mediated activation. This shift can be overactive and in some reported cases the activity level does not return to the basal level. The abnormal higher activity from the heterozygous 838 mutations leads to CRD, while the loss of partial or total function is tolerable in heterozygous status but is causative and leads LCA in biallelic status (Sharon et al., 2018). For *RP1* truncation variants, some studies have excluded haploinsufficiency and gain-of-function as the causative mechanism of *RP1* variants (Liu et al., 2012; Nanda et al., 2019). The variants involving the BIF domain, which is crucial for the photoreceptor (Bahri et al., 1997; Pierce et al., 1999), will lead to haploinsufficiency of *RP1* either by triggering nonsense-mediated decay or by producing a loss-of-function protein. Therefore, heterozygous variants located within or upstream of the BIF domain will not cause diseases. For the variants associated with adRP, the truncated production will preserve the BIF domain and may cause disease via a dominant-negative effect (Chen et al., 2010; Nanda et al., 2019). However, the variants at the C-terminal recessive region, which can also produce a protein with the BIF domain, would not cause diseases in heterozygous status. It has been assumed that an unrecognized domain is present downstream of the BIF domain (Baum et al., 2001). The unknown domain may be important for the interaction of *RP1* with other proteins by cooperating with the BIF domain. It implies that the heterozygous variants will be non-pathogenic either loss of both the BIF domain and the unrecognized domain or preserve with both domains, while the heterozygous variants will cause retinal degeneration with preserved BIF domain but loss of the unrecognized domain. Functional studies are expected to disclose the exact mechanism of the unique feature associated with *RP1* variants.

Genotype–phenotype analysis revealed that patients with biallelic variants showed more severe phenotypes than those with heterozygous variants, including an earlier age at onset, worse visual acuity, and fundus changes especially in the macular region. The limitation of this study is not knowing the exact age at onset because of the nature of a retrospective study and lack of supporting evidence from functional studies.

In conclusion, in this study, a pooled analysis of our exome-sequencing data and the literature review confirmed and refined the common features and the boundaries between dominant and recessive truncation variants in *RP1.* It also raises unsolved problems that are worth investigating in the future. The unique features and questions identified in *RP1* may not only be valuable for its clinical application and further studies but also reminds us of the possibility of such features and questions in other genes that are awaited to be identified.

## Data Availability Statement

The raw data supporting the conclusions of this article will be made available by the authors, without undue reservation.

## Ethics Statement

The studies involving human participants were reviewed and approved by the institutional review board of the Zhongshan Ophthalmic Center. Written informed consent to participate in this study was provided by the participants’ legal guardian/next of kin.

## Author Contributions

XX, SL, and QZ recruited the individuals diagnosed with different forms of eye conditions. XX, JW, and QZ collected the clinical records. XX, SL, PW, and QZ performed the whole-exome analysis and targeted-exome sequencing. JW, WS, and QZ performed the bioinformatic analysis and designed the study and discussed the results and wrote the manuscript. JW and WS confirmed the variants by Sanger sequencing and did the statistical analysis of clinical data. JW, WS, and QZ discussed the results and wrote the manuscript. All authors reviewed and approved the manuscript.

## Conflict of Interest

The authors declare that the research was conducted in the absence of any commercial or financial relationships that could be construed as a potential conflict of interest.

## Footnotes

1 https://gnomad.broadinstitute.org/

2 https://sites.google.com/site/revelgenomics/

3 https://cadd.gs.washington.edu/

4 http://sift.jcvi.org/www/SIFT_enst_submit.html

5 http://genetics.bwh.harvard.edu/pph2/index.shtml

6 http://provean.jcvi.org

7 http://www.fruitfly.org/

8 http://primer3.ut.ee/

9 https://www.ncbi.nlm.nih.gov/pubmed/

10 http://so.hiqq.com.cn/

11 http://www.hgmd.cf.ac.uk/ac/index.php

## Supplementary Material

Supplementary Figure 1 Pedigrees and Sanger sequencing chromatography of unrelated families with identified *RP1* variants in this study. For each family, the pedigree is shown in the left column and the sequence changes are shown in the right column. Mx, mutant allele; +, wild-type allele.

Supplementary Figure 2 The distributions and frequencies of the missense variants in *RP1*. The positions and allele counts of the heterozygous *RP1* variants are displayed above, while those of the biallelic *RP1* variants are displayed below. RP, retinitis pigmentosa; CRD, cone-rod dystrophy; MD, macular degeneration; IRD, inherited retinal disease; LCA, Leber congenital amaurosis; STGD, Stargardt disease. **DCX domain:** c.106–354 (p.36–188) and c.460–699 (p.154–233). BIF domain: c.1456-1959 (p.486-653). **(A)** Distributions and frequencies of the missense variants in the gnomAD database. **(B)** Distributions and frequencies of the missense variants in the published literature. **(C)** Distributions and frequencies of the missense variants in this study.

Supplementary Table 1 Clinical information of probands with *RP1* variants in our cohort.

Supplementary Table 2 Biallelic variants in *RP1* not associated with RP.

Supplementary Table 3 Single heterozygous truncation variants in *RP1* that were not associated with RP in our cohort.

Supplementary Table 4 Truncation *RP1* variants in published literature.

Supplementary Table 5 All missense *RP1* variants in published literature.

Supplementary Table 6 Truncation variants in *RP1* reported in patients with conflict pattern of inheritance.
