# Supplementary figures and images for: Dominant RP in the Middle While Recessive in Both the N- and C-Terminals Due to RP1 Truncations: Confirmation, Refinement, and Questions
Source: Front Cell Dev Biol. 2021 Feb 19;9:634478. doi: 10.3389/fcell.2021.634478 (PMC7935555; doi:10.3389/fcell.2021.634478)

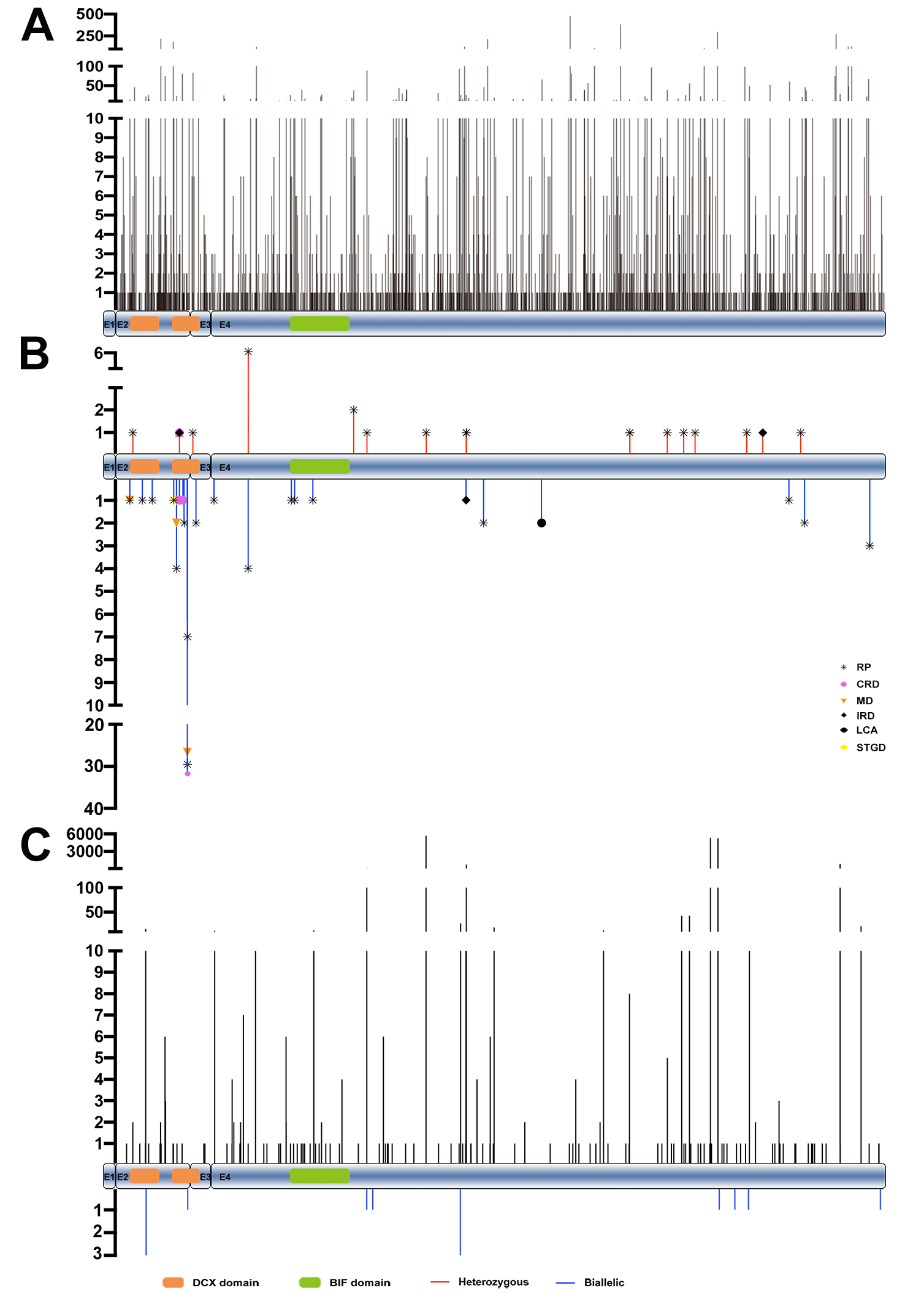

Supplement: Supplementary Figure 1 — Pedigrees and Sanger sequencing chromatography of unrelated families with identified RP1 variants in this study. For each family, the pedigree is shown in the left column and the sequence changes are shown in the right column. Mx, mutant allele; +, wild-type allele. [file Image_1.TIF]
