# Supplementary material for: Dominant RP in the Middle While Recessive in Both the N- and C-Terminals Due to RP1 Truncations: Confirmation, Refinement, and Questions
Source: Front Cell Dev Biol. 2021 Feb 19;9:634478. doi: 10.3389/fcell.2021.634478 (PMC7935555; doi:10.3389/fcell.2021.634478)
Supplement: Supplementary Figure 2 — The distributions and frequencies of the missense variants in RP1. The positions and allele counts of the heterozygous RP1 variants are displayed above, while those of the biallelic RP1 variants are displayed below. RP, retinitis pigmentosa; CRD, cone-rod dystrophy; MD, macular degeneration; IRD, inherited retinal disease; LCA, Leber congenital amaurosis; STGD, Stargardt disease. DCX domain: c.106–354 (p.36–188) and c.460–699 (p.154–233). BIF domain: c.1456-1959 (p.486-653). (A) Distributions and frequencies of the missense variants in the gnomAD database. (B) Distributions and frequencies of the missense variants in the published literature. (C) Distributions and frequencies of the missense variants in this study. [file Data_Sheet_1.PDF]

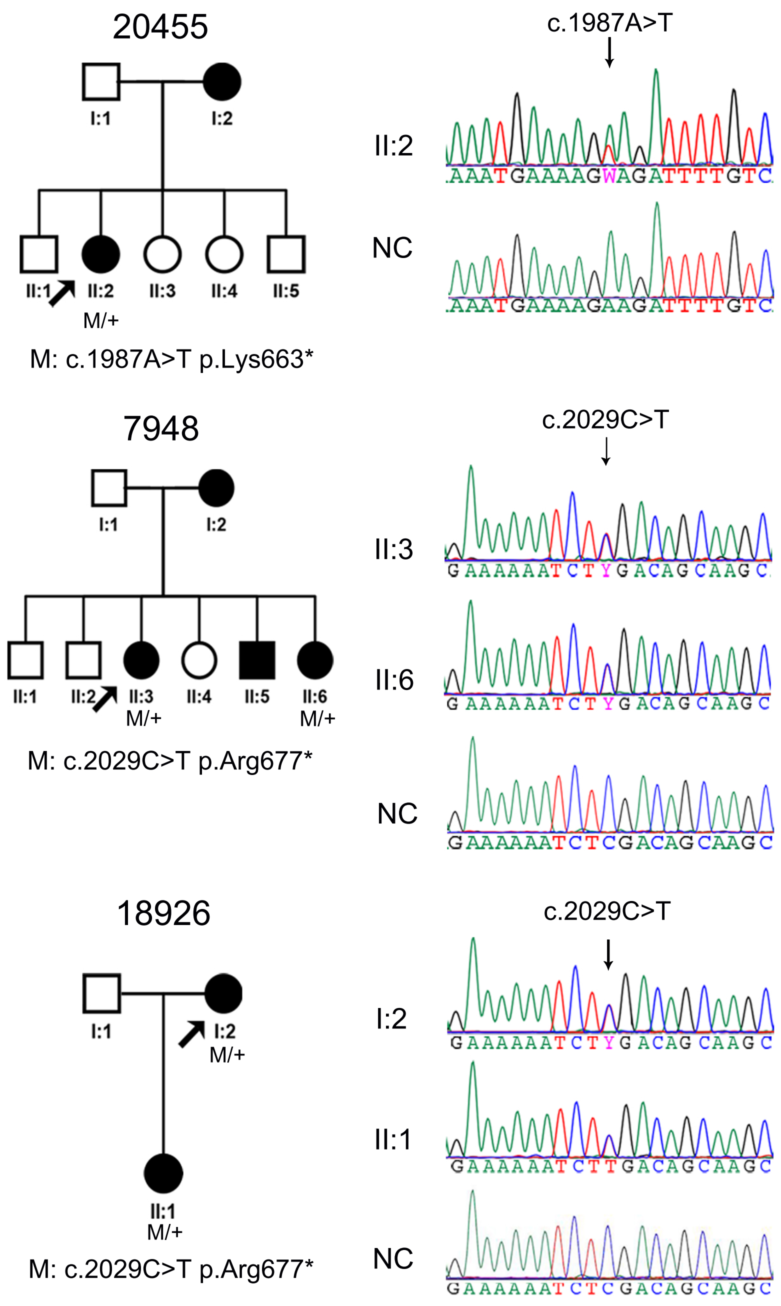
20455
I:1
I:2
II:1
II:2
II:3
II:4
II:5
M/+
M: c.1987A>T p.Lys663*
c.1987A>T
II:2
AAATGAAAAGWAGATTTTGTC
NC
AAATGAAAAGAAGATTTTGTC
7948
I:1
I:2
II:1
II:2
II:3
II:4
II:5
II:6
M/+
M/+
M: c.2029C>T p.Arg677*
c.2029C>T
II:3
GAAAAAATCTYGACAGCAAGC
II:6
GAAAAAATCTYGACAGCAAGC
NC
GAAAAAATCTCGACAGCAAGC
18926
I:1
I:2
M/+
II:1
M/+
M: c.2029C>T p.Arg677*
c.2029C>T
I:2
GAAAAAATCTYGACAGCAAGC
II:1
GAAAAAATCTTGACAGCAAGC
NC
GAAAAAATCTCGACAGCAAGC

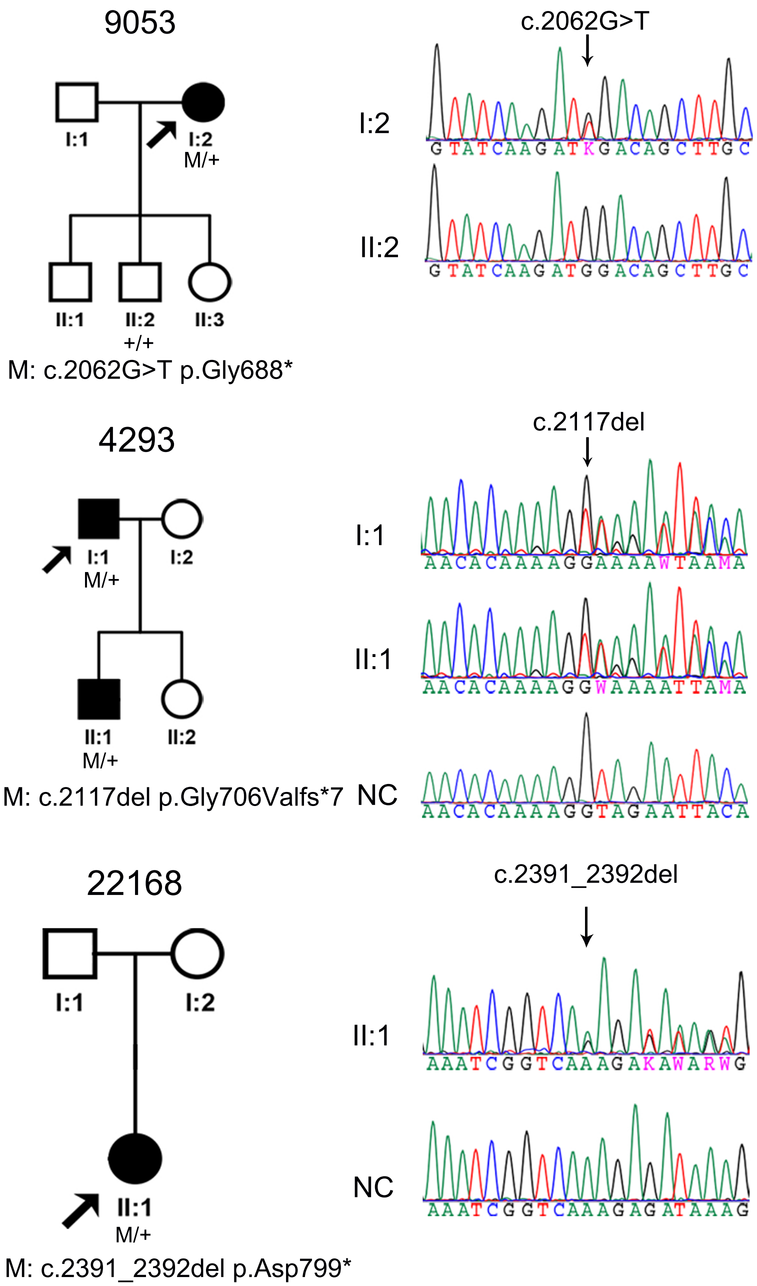
9053
I:1
I:2
M/+
II:1
II:2
+/+
II:3
M: c.2062G>T p.Gly688*
c.2062G>T
I:2
GTATCAAGATKGACAGCTTGC
II:2
GTATCAAGATGGACAGCTTGC
4293
I:1
M/+
I:2
II:1
M/+
II:2
M: c.2117del p.Gly706Valfs*7
c.2117del
I:1
AACACAAAAGGAAAAWTAAMA
II:1
AACACAAAAGGWAAAATTAMA
NC
AACACAAAAGGTAGAATTACA
22168
I:1
I:2
II:1
M/+
M: c.2391_2392del p.Asp799*
c.2391_2392del
II:1
AAATCGGTCAAAGAKAWARWG
NC
AAATCGGTCAAAGAGATAAAG

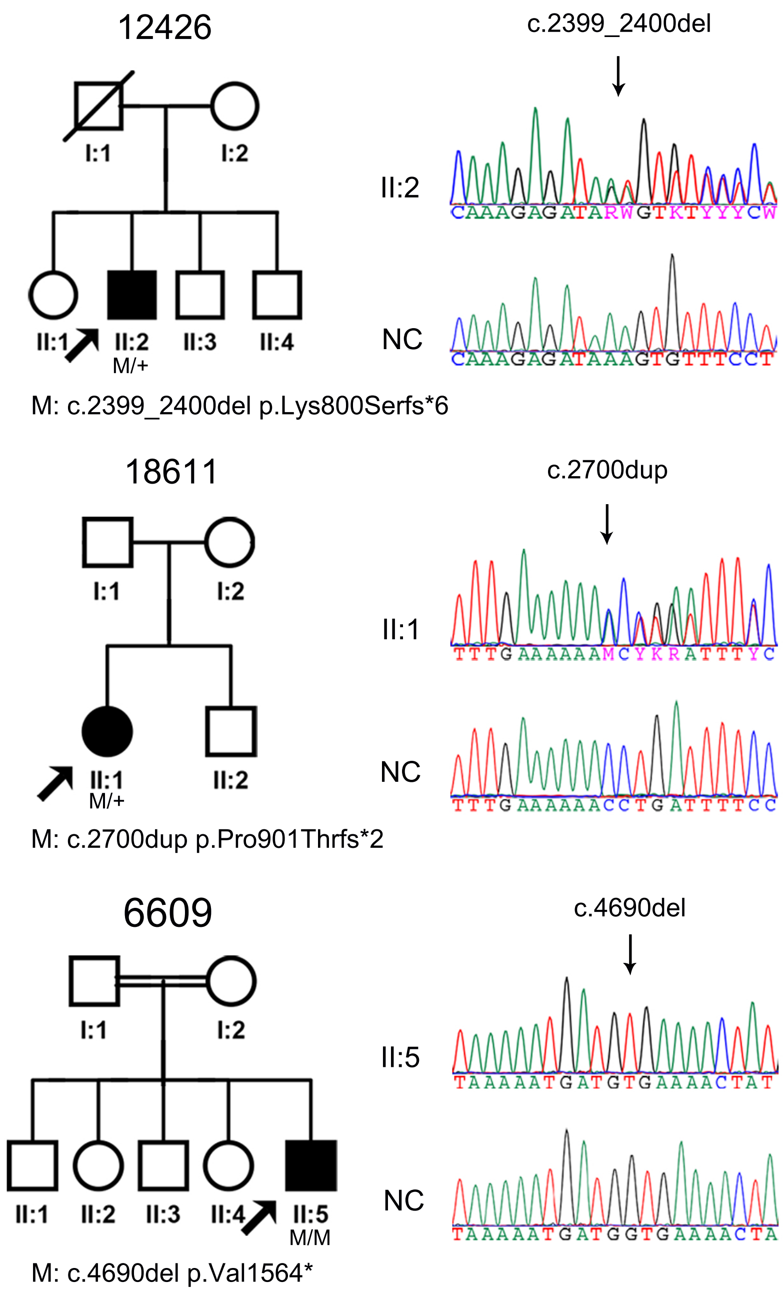
12426
c.2399_2400del
I:1
I:2
II:2
CAAAGAGATARWGTKTYYYCW
NC
CAAAGAGATAAAGTGTTTCCT
II:1
II:2
II:3
II:4
M/+
M: c.2399_2400del p.Lys800Serfs*6
18611
c.2700dup
I:1
I:2
II:1
TTTGAAAAAAMCYKRATTTYC
NC
TTTGAAAAAACCTGATTTTCC
II:1
M/+
II:2
M: c.2700dup p.Pro901Thrfs*2
6609
c.4690del
I:1
I:2
II:5
TAAAAATGATGTGAAAACTAT
NC
TAAAAATGATGGTGAAAACTA
II:1
II:2
II:3
II:4
II:5
M/M
M: c.4690del p.Val1564*

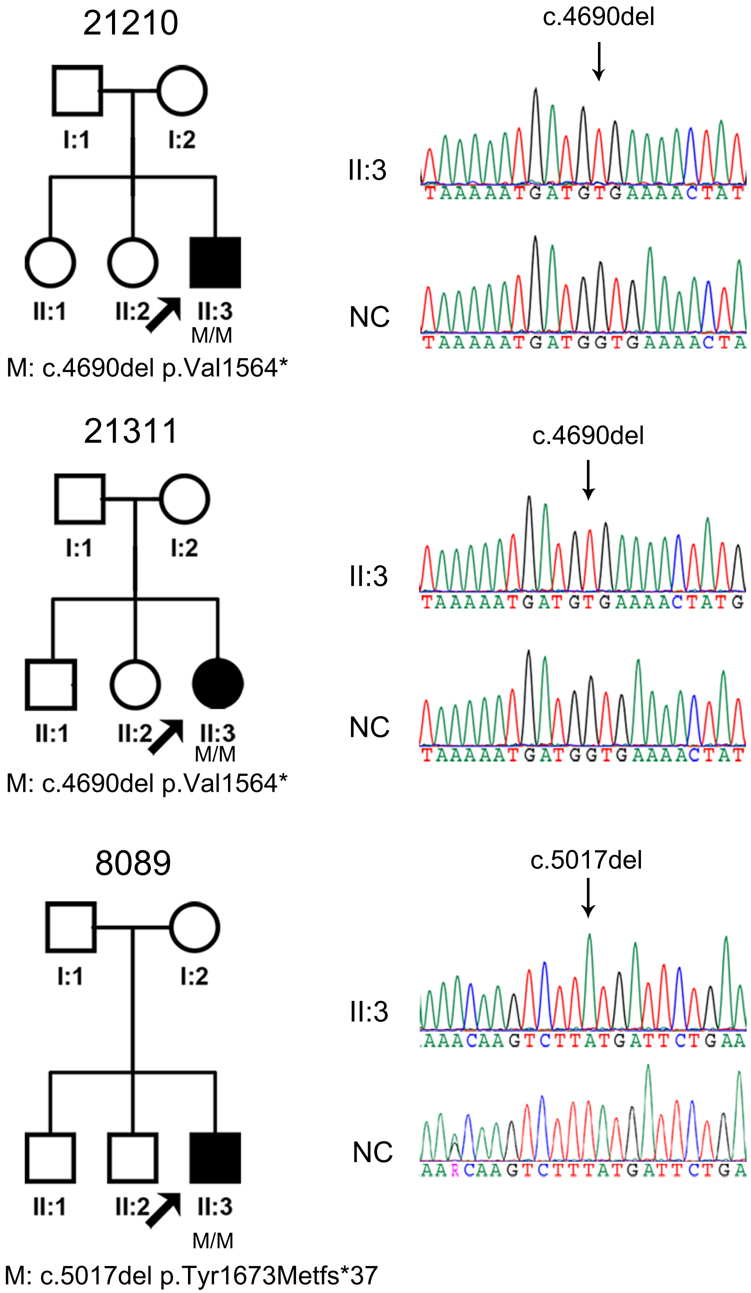
21210
c.4690del
I:1
I:2
II:3
II:1
II:2
II:3
M/M
NC
M: c.4690del p.Val1564*
21311
c.4690del
I:1
I:2
II:3
II:1
II:2
II:3
M/M
NC
M: c.4690del p.Val1564*
8089
c.5017del
I:1
I:2
II:3
II:1
II:2
II:3
M/M
NC
M: c.5017del p.Tyr1673Metfs*37

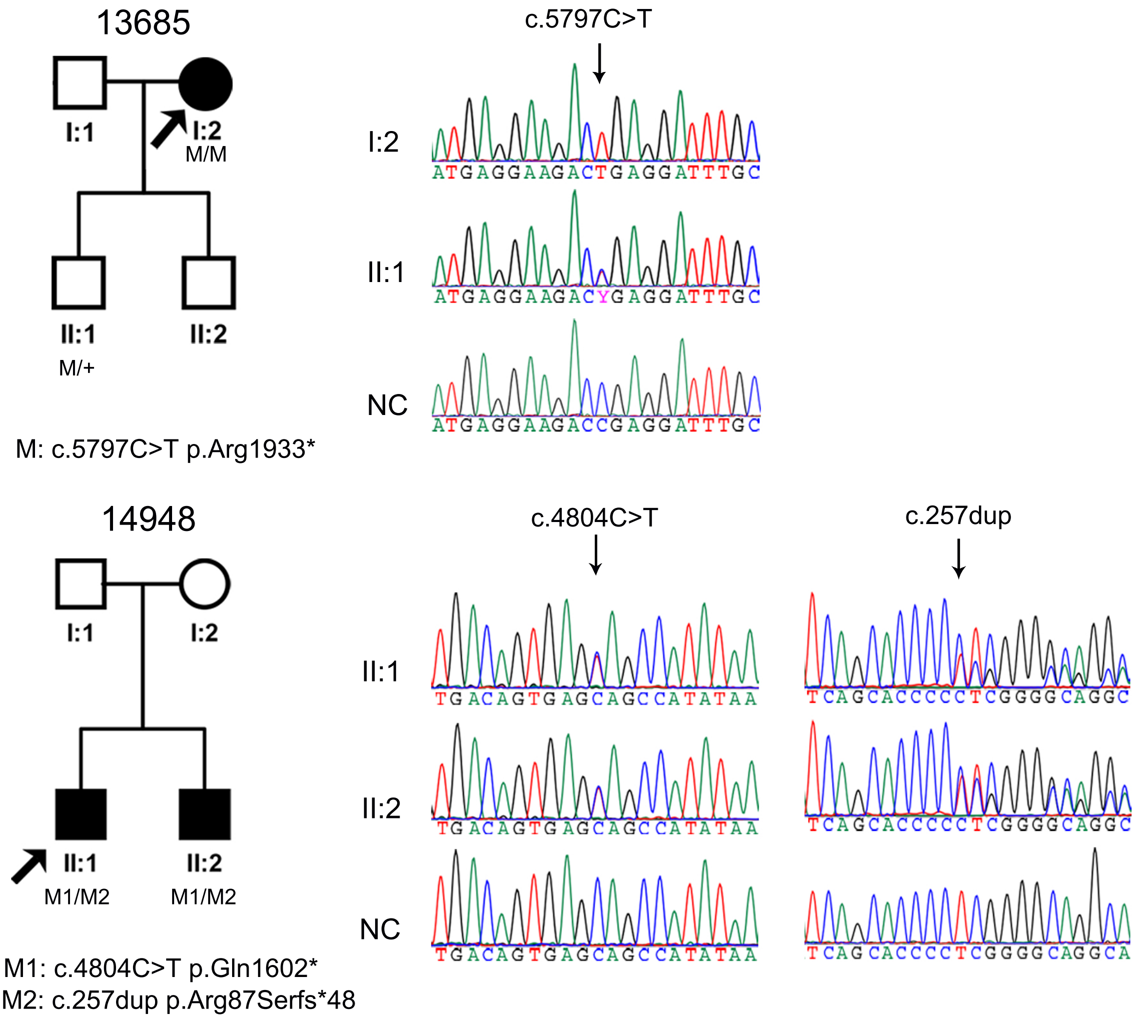
13685
I:1
I:2
M/M
II:1
II:2
M/+
M: c.5797C>T p.Arg1933*
c.5797C>T
I:2
ATGAGGAAGACTGAGGATTTGC
II:1
ATGAGGAAGACYGAGGATTTGC
NC
ATGAGGAAGACCGAGGATTTGC
14948
I:1
I:2
II:1
II:2
M1/M2
M1/M2
M1: c.4804C>T p.Gln1602*
M2: c.257dup p.Arg87Serfs*48
c.4804C>T
c.257dup
II:1
TGACAGTGAGCAGCCATATAA
TCAGCACCCCCTCGGGGCAGGC
II:2
TGACAGTGAGCAGCCATATAA
TCAGCACCCCCTCGGGGCAGGC
NC
TGACAGTGAGCAGCCATATAA
TCAGCACCCCTCGGGGCAGGCA
